# Current topics in the surgical treatments for hepatocellular carcinoma

**DOI:** 10.1002/ags3.12065

**Published:** 2018-02-28

**Authors:** Daisuke Ban, Toshiro Ogura, Keiichi Akahoshi, Minoru Tanabe

**Affiliations:** ^1^ Department of Hepatobiliary and Pancreatic Surgery Graduate School of Medicine Tokyo Medical and Dental University Tokyo Japan

**Keywords:** hepatectomy, hepatocellular carcinoma, liver resection, surgical treatment

## Abstract

Treatment strategy for hepatocellular carcinoma (HCC) requires optimal selection of therapies based on various factors related to tumor condition and liver functional reserve. Although several evidence‐based guidelines have been proposed for the treatment of HCC, the criteria and range of indications differ among these guidelines according to the circumstances of each country. In European nations and the USA, patients with the Barcelona Clinic Liver Cancer stage 0‐A are subjects for surgical resection, whereas in Asian countries, even those with the intermediate stage are regarded as surgical candidates. Furthermore, since the recent introduction and rapidly widely spreading use of laparoscopic liver resection, this technique has become an important treatment option for surgical resection. In this review article, we overview the current topics of treatment of HCC with a special focus on surgical therapy.

## Abstract 2

Overview of the surgical treatments for hepatocellular carcinoma

## 1 INTRODUCTION

Approximately 850 000 people die of hepatocellular carcinoma (HCC) per year, worldwide^1^, ^2^ and it is the second most common cause of cancer death. Approximately half of all primary liver cancers occur in China (395 000 people per year), whereas Northern Europe has the lowest incidence of the tumor.^3^ The incidence of hepatocellular carcinoma has increased in many countries, depending on hepatitis B and C virus infection and alcohol‐related liver disease. Other risk factors include smoking, obesity, nonalcoholic fatty liver disease (NAFLD) and diabetes.^4^

Resection is the mainstay of treatment for resectable HCC.^5^, ^6^ In addition to the surgical treatments, there are various therapeutic options, including locoregional treatment, transcatheter arterial chemoembolization (TACE), and systemic treatment. Moreover, the background of HCC treatment differs widely according to the condition of each institution and availability of donors for transplantation. We review the current topics of HCC treatment regarding the position of surgical treatments from the standpoints of comparison of guidelines, early‐stage HCC and advanced‐stage HCC. We also discus laparoscopic liver resection, a surgical technique that has become popular recently.

## 2 CURRENT STATUS OF THE TREATMENT GUIDELINES FOR HEPATOCELLULAR CARCINOMA

Although a number of staging systems for HCC have been established, no single universal staging system exists that could be applicable to all patients in various countries. The two major systems are the tumor, node, metastasis (TNM) system of the American Joint Committee on Cancer (AJCC)/Union for International Cancer Control (UICC), and the Barcelona Clinic Liver Cancer (BCLC) system.^7^, ^8^

### 2.1 TNM staging

The TNM staging system is based on three key pieces of information. T describes the number and size of the primary tumor(s), and whether the tumor has grown into nearby blood vessels. N describes the extent of spread to regional lymph nodes. M indicates whether the tumor has metastasized to distant parts of the body. The most recent version of the TMN staging system (8th edition) was published in 2017, coming into effect on 1 January 2018.^7^ Some significant changes in the T classification have been made, relative to the 7th edition. T1 was subdivided into two subcategories: T1a (solitary tumor ≤2 cm) and T1b (solitary tumor >2 cm, without vascular invasion). There was no change to the T2 category (solitary tumor with vascular invasion or multiple tumors, none >5 cm). The previous T3a category was re‐categorized as T3 (multiple tumors, at least one of which is >5 cm), whereas tumors that were previously categorized as T3b are now included in T4 (tumors involving a major branch of the portal vein or hepatic vein, or tumors with direct invasion of adjacent organs or perforation of the visceral peritoneum).

Because the TNM staging system lacks factors related to liver functional reserve, it may not be adequate for patients with severe underlying liver disease.^9^

### 2.2 BCLC staging classification

The BCLC group was created in 1986 by Jordi Bruix and Concepcio Bru. Since the staging system was first published in 1999, it has been updated according to evidence‐based data.^8^

The BCLC staging system comprises five stages that are based on the extent of the primary lesion, performance status, liver function, vascular invasion and extrahepatic metastasis. BCLC defines very early‐stage cancer (<2 cm, single nodule, Child‐Pugh A) as stage 0 and cases involving a single nodule or <3 nodules of <3 cm as early stage (stage A). Multinodular HCC is defined as intermediate stage (stage B), cases involving vascular invasion or extrahepatic spread as advanced stage (stage C), and cases where the patient has Child‐Pugh C cirrhosis or a performance status >2 as terminal stage (stage D). According to the most recent version of their treatment algorithm, patients with BCLC stage 0 or stage A can benefit from potentially curative treatments, including resection, transplantation and ablation.^10^ Patients with HCC of BCLC stage B are considered for TACE. However, patients with a great tumor burden (size >10 cm) or impairment of liver function are not good candidates for TACE. The first‐line treatment option for patients with BCLC stage C is sorafenib.^11^

### 2.3 Comparison of indications for surgical treatment in the current guidelines

Since the year 2001, when the European Association for the Study of the Liver (EASL) published HCC management guidelines,^12^ more than 20 guidelines have been published and each has its own characteristics (Table 1).^13^

**Table 1:** Comparison of the indications for hepatic resection

Region/ Year	Guidelines		Liver function factor			Tumor factor		Comment
			Child‐Pugh	Portal hypertension	ICG test	Size and number	Vascular invasion	
Europe								
2012	EASL‐EORTC	European Association for Study of Liver, European Organization for Research and Treatment of Cancer	A	No PH	(+)	Single	No VI	Based on BCLC
2012	ESMO‐ESDO	European Society for Medical Oncology	A	No PH	(−)	Single, size ≤5 cm or ≤3 nodules, size ≤3 cm	No VI	Based on BCLC
USA								
2017	AASLD	American Association for the Study of Liver Disease	A	Minimal/no PH	(−)	Single, size ≤5 cm or ≤3 nodules, size ≤3 cm	No VI	
2017	NCCN	National Comprehensive Cancer Network	A, B	Mild/no PH	(−)	UNOS criteria	No major VI	Based on resectability
Asia								
2012	Chinese	Primary Liver Cancer Diagnosis and Treatment Expert Panel of the Chinese Ministry of Health	A	(−)	(+)	≤3 nodules	Limited intrahepatic	Choice of palliative hepatectomy
2012	Saudi	Saudi Association for the Study of Liver Disease and Transplantation; Saudi Oncology Society	A, early B	No PH	(−)	Multinodular (resectable)	No VI	Based on BCLC
2014	INASL	The Indian National Association for Study of the Liver	A, B	Mild/no PH	(+)	Single or ≤3 nodules, size ≤3 cm	No VI	Based on BCLC
2015	JSH	Japan Society of Hepatology	A, B	(−)	(+)	≤3 nodules	If resectable	Vascular invasion is not excluded
2015	KLCSG	Korean Liver Cancer Study Group and National Cancer Center	A, superb B	Mild/no PH	(+)	≤3 nodules	No VI	mUICC staging system
2017	APASL	Asian Pacific Association for the Study of the Liver	A, B	(−)	(+)	Resectable	No VI	Resectability should be judged by a multidisciplinary team

United Network for Organ Sharing (UNOS) criteria, single lesion ≤5 cm, or 2 or 3 nodules ≤3 cm; BCLC, Barcelona Clinic Liver Cancer; ESDO, European Society of Digestive Oncology; ICG, indocyanine green retention test; mUICC, modified UICC; PH, portal hypertension; VI, vascular invasion; (−), excluded as a factor; (+), included as a factor.

In general, indication for hepatic resection is decided based on liver function and extent of tumor development. Appropriate candidates for surgical treatment vary according to the guidelines.

### 2.4 Liver function

Liver function is assessed on the basis of the Child‐Pugh classification, the presence of portal hypertension, such as thrombocytopenia associated with varicose veins and splenomegaly, and the presence of elevated serum bilirubin concentrations. According to the BCLC staging, criteria for surgical candidates include those classified as Child‐Pugh class A, absence of portal hypertension, and elevated bilirubin.^10^ The criteria set by the American Association for the Study of Liver Disease (AASLD) and EASL‐European Organization for Research and Treatment of Cancer (EORTIC) are identical to those in the BCLC staging.^14^, ^15^ However, the European Society of Medical Oncology, and European Society of Digestive Oncology (ESMO‐ESDO) guidelines exclude portal hypertension from the treatment algorithm, thereby widening the surgical indication.^15^ According to the National Comprehensive Cancer Network (NCCN), Japanese, and Korean guidelines, it is recommended not to consider mild portal hypertension as a contraindication to surgical treatments. It is evident that clinically significant portal hypertension increases the risk of postoperative mortality and clinical decompensation.^16^ However, further investigation is required to determine the severity of portal hypertension, which may not impair the safety of surgery. It has been reported that portal hypertension may not affect the prognosis after radiofrequency ablation (RFA).^17^ In the Japanese and Korean guidelines, liver functional reserve is assessed by the indocyanine green (ICG) test, in addition to the Child‐Pugh classification system.^18^, ^19^

### 2.5 Tumor factors

Staging of tumors is determined on the basis of size and number of tumors, presence or absence of vascular invasion, and presence or absence of extrahepatic lesions. In the EASL‐EORTC and ESMO‐ESDO guidelines from Europe, and the Saudi and INASL (India) guidelines from Asia, treatment strategy is determined in accordance with the BCLC staging system. In general, cases with a solitary tumor without vascular invasion are indicated for surgical resection. However, in the ESMO‐ESDO, Saudi, and INASL guidelines, the indication is extended slightly to include cases with multiple tumors (BCLC stage A).^5^, ^15^, ^20^, ^21^

The EASL‐EORTC guidelines added a recommendation of anatomical resection that has ensured a surgical approach, based on sound oncological principles, although associated with a modest decrease in early recurrence.^22^, ^23^ The guidelines also refer to the choice of preoperative portal vein embolization (PVE) in order to increase the residual liver volume if a major resection is planned.^5^

The AASLD guidelines were based on the BCLC staging system in the previous version (2011).^14^ However, unlike previous AASLD guidelines, the new practice guidelines published in 2017 were developed in compliance with the Institute of Medicine for trustworthy practice guidelines and use the Grading of Recommendation Assessment, Development and Evaluation (GRADE) approach.

The current AASLD guidelines revised the candidates for surgical therapy and recommend that resectable T1 and T2 tumors are indicated for resection. Resectable HCC is defined as those: (i) with one to three unilobar lesions, with an upper size limit of 5 cm for single lesions and 3 cm for more than one lesion; (ii) without extrahepatic disease or macrovascular invasion; and (iii) occurring in the setting of minimal or no portal hypertension and in the absence of synthetic dysfunction (BCLC 0 or A). At the same time, the AASLD emphasized that a number of clinical and laboratory variables, including the availability of alternative therapies, can influence the individual clinician's decision to proceed with resection. Also, they mentioned that a new guidance document had been created and would be published soon; therefore, we need to focus on their future trends.^20^

Treatment algorithms in the NCCN and the Asian Pacific Association for the Study of Liver (APASL) guidelines are based on tumor resectability. The APASL guideline recommends that, when judging resectability, both technical and oncological aspects should be taken into consideration.^24^ In the NCCN guidelines, cases of a single nodule without vascular invasion are considered the best candidates for surgery, whereas cases matching the United Network for Organ Sharing (UNOS) criteria (single lesion ≤5 cm, or 2 or 3 lesions ≤ 3 cm) are candidates for both liver transplantation and surgical resection.^25^

The Korean guidelines have adopted the fifth version of the modified International Union for Cancer Control (UICC) staging system. Based on this system, the Korean guidelines state that resection can be considered in patients with ≤3 nodules without vascular invasion.^19^

The Japanese guidelines also recommend that resection can be considered in patients with ≤3 nodules, regardless of tumor size. In addition, portal vein tumor thrombus should not be precluded from surgical resection, so that these guidelines recommend the widest surgical indication.^18^ However, the newly updated Japan Society of Hepatology (JSH) guidelines recommend that the optimal treatment strategy for HCC with vascular invasion should be selected from TACE, liver resection, hepatic arterial infusion therapy, and molecular targeted drugs, according to the conditions of individual cases.^26^

The Chinese guidelines define general surgical indication for cases with three or fewer tumors, meanwhile proposing palliative liver resection and seeking potential surgical therapy for advanced cases, including resection of portal vein tumor thrombus (PVTT) and concomitant splenectomy for cases with portal hypertension.^27^

### 2.6 Liver transplantation

Liver transplantation is an appropriate strategy for patients with localized HCC who are not candidates for resection. The Milan criteria, which were established by Mazzaferro in 1996, have been applied widely around the world in the selection of patients for liver transplantation.^28^ The Milan criteria are too strict in terms of post‐transplant recurrence rates and could be expanded, as long as patient outcome is not impaired. The University of California, San Francisco (UCSF) criteria are the most widely accepted for the expansion of the Milan criteria: solitary tumor <65 mm, or two to three tumors <45 mm and total tumor diameter <80 mm, without vascular invasion or distant metastasis.^29^ Excessive expansion of inclusion criteria will result in an increase in waiting time and a deterioration of survival among patients who are on the waiting list, not only those with HCC but also those without. According to a study by the US transplant registry, expansion of the Milan criteria will require a 5‐year survival rate of 61% after transplantation to prevent a substantial decrease in the survival of other patients on the waiting list.^30^ In Asian countries, where living‐donor liver transplantation (LDLT) accounts for the majority of liver transplantations, the indication is different to some degree. Unlike deceased‐donor transplantation, LDLT is not limited by the restrictions imposed by a nationwide organ allocation system. Thus, the indication for LDLT depends on case‐by‐case considerations, balancing the burden on the donor, the operative risk and the survival benefit.^31^

On the waiting list for transplantation, HCC patients can experience tumor growth and drop‐out from the waiting list. Belghiti et al^32^ showed that liver resection prior to liver transplantation for HCC does not impair survival following transplantation, and mentioned that resection could be used as a bridge therapy to transplantation. Liver resection and liver transplantation should be associated, rather than considered separately.

## 3 CURRENT TREATMENTS FOR HCC

### 3.1 Treatments for HCC at an early stage

#### 3.1.1 The position of resection

Regarding the resectability of HCC, there are two standpoints, technical resectability and oncological resectability. The technical point of view represents the possibility of resecting a tumor safely without complication, whereas the oncological point of view is that the resection should be non‐inferior or superior to other treatment methods. However, it is very difficult to define “satisfactory prognosis”, which can justify the invasiveness of surgical procedures.^24^ In addition, inclusion of liver transplantation as one of the treatment options may further complicate the situation because of the problem of availability of donors, as well as the involvement of social, economic, and ethical viewpoints.^33^

Because the BCLC recommends a narrower surgical indication, strictly conforming to these guidelines could fail to decide the optimal treatment for patients. In fact, 50% of patients with intermediate‐ to advanced‐stage disease defined by the BCLC routinely underwent surgical resection.^34^ In contrast, only 10% to 35% of patients with very early‐ to early‐stage disease underwent liver resection because of small residual liver volume, low liver functional reserve, or not good performance status, although resection is recommended as a first‐line treatment on BCLC.^14^, ^35^ Those patients who are not suitable for surgical indication are the best candidates for ablation therapy, including RFA. The Hong Kong guidelines were published in 2016 with a wider surgical indication than the BCLC.^36^

#### 3.1.2 Resection versus RFA

Among the various ablation therapies, RFA is the most popular; other modalities, including ethanol injection and acetic acid injection as chemical ablation, microwave ablation, laser ablation, cryoablation, high‐intensity focused ultrasound and irreversible electroporation as thermal ablation are also used.

Many retrospective observational studies regarding surgical resection and ablation for early‐stage HCC have been published and most have reported the superiority of resection.^37^, ^38^

There have been many retrospective studies that compare resection and ablation using propensity score matching. Liu et al^39^ compared resection and RFA and found no difference in overall survival (OS) but better prognosis was observed for the resection group in terms of disease‐free survival (DFS). According to a report from Lee et al, both resection and RFA showed better prognosis than TACE for patients with BCLC stage A HCC, but there was no difference in survival between resection and RFA.^40^ Chong et al^41^ showed the superiority of resection over RFA in terms of OS and DFS in patients with BCLC stage 0/A HCC.

Four randomized controlled trials (RCT) have been reported so far.^42^, ^43^, ^44^, ^45^ Huang et al^43^ compared the outcome between resection and RFA in patients with HCC which met the Milan criteria, and showed the superiority of resection (Table 2). However, the conclusion stating the superiority of resection to RFA cannot be accepted completely because some studies included patients with HCC of 2 cm or greater in size, for which ablation seems ineffective, and because RFA was more likely to be selected for patients with impaired liver functional reserve, so that there may be a difference in patient background, suggesting the possibility of selection bias.^39^, ^48^

**Table 2:** Recent RCT and PSM studies regarding outcomes of liver resection vs radiofrequency ablation for HCC

Author	Year	Type	Treatments	Liver function factor	Tumor factor	Survival	RFS
Chen et al^42^	2006	RCT	LR (n = 90) vs RFA (n = 71)	CP‐A ICG‐R15 <30% Plt >40 000/mm^3^	Single Size ≤5 cm	NSD LR 3 y 73.4% RFA 3 y 71.4%	NSD LR 3 y 69.0% RFA 3 y 64.1%
Huang et al^43^	2010	RCT	LR (n = 115) vs RFA (n = 115)	CP‐A and B ICG‐R15 <20% Plt >50 000/mm^3^	Within Milan criteria	LR > RFA LR 3 y 92.2% RFA 3 y 69.6%	LR > RFA LR 3 y 60.9% RFA 3 y 46.1%
Feng et al^44^	2012	RCT	LR (n = 84) vs RFA (n = 84)	CP‐A and B ICG‐R15 <30% Plt >50 000/mm^3^	Single/2 nodules Size ≤4 cm	NSD LR 3 y 74.8% RFA 3 y 67.2%	NSD LR 3 y 61.1% RFA 3 y 49.6%
Fang et al^45^	2014	RCT	LR (n = 60) vs RFA (n = 60)	CP‐A and B Plt >50 000/mm^3^	Single/2/3 nodules Size ≤3 cm	NSD LR 3 y 77.5% RFA 3 y 82.5%	NSD LR 3 y 41.3% RFA 3 y 55.4%
Jiang et al^46^	2015	PSM	LR (n = 140) vs RFA (n = 140)	BCLC stage A	Single/2 nodules Size ≤3 cm	NSD LR 3 y 74.8% RFA 3 y 72.9%	LR > RFA LR 3 y 52.4% RFA 3 y 35.8%
Kim et al^47^	2016	PSM	LR (n = 152) vs RFA (n = 152)	PS 0, CP‐A	Single nodules Size ≤3 cm	NSD	NSD
Liu et al^39^	2016	PSM	LR (n = 79) vs RFA (n = 79)	BCLC stage 0	Single Size ≤ 2 cm	NSD LR 3 y 97% RFA 3 y 88%	LR > RFA LR 3 y 64% RFA 3 y 38%
Chong et al^41^	2017	PSM	LR (n = 121) vs RFA (n = 121)	BCLC stage 0/A	Single/2/3 nodules Size ≤3 cm	LR > RFA LR 3 y 90% RFA 3 y 76%	LR > RFA LR 3 y 63% RFA 3 y 30%

BCLC, Barcelona Clinic Liver Cancer; CP, Child‐Pugh; HCC, hepatocellular carcinoma; ICG, indocyanine green retention test; LR, liver resection; NSD, no significant difference; Plt, platelets; PS, performance status; PSM, propensity‐score matched study; RCT, randomized controlled trial; RFA, radiofrequency ablation.

Indeed, in daily clinical practice, treatment regimens are determined not only by the size and number of tumors but also by the location of tumors and their relationship to blood vessels, as well as the liver functional reserve of patients with Child A classification. Some reports also discussed the cost‐benefit of treatments, in addition to tumor factors and liver functional reserve, and suggested that the optimal treatment should be considered not only based on tumor factors defined in the guidelines but also on more detailed conditions.^49^, ^50^

### 3.2 Treatments for HCC at an advanced stage

HCC with vascular invasion is classified as advanced stage according to the BCLC algorithm and treatment with sorafenib is recommended.^14^ In addition, the consensus guidelines of the APASL recommend clinical study of sorafenib or systemic chemotherapy for HCC with tumor thrombus extending to the major branch of the portal vein.^51^ However, the outcome of sorafenib treatment for advanced HCC does not seem favorable.^52^ We will discuss surgical treatment for advanced HCC, compared with transarterial chemoembolization, adjuvant therapy, and systemic chemotherapy.

#### 3.2.1 Surgical treatments for HCC at an advanced stage

Jiang et al^53^ reviewed the outcomes of surgical resection of HCC associated with macroscopic PVTT and reported a median survival time of 9 to 64 months, with some variability among studies. Kokudo et al^54^ analyzed 6474 cases of HCC with PVTT in a Japanese national survey from 2000 to 2007. In cases with HCC having PVTT extending to the first branch of the portal vein (Vp1‐3), the prognosis was significantly better in patients who underwent liver resection than in those who underwent non‐surgical treatments, and a similar result was obtained from a propensity score‐matched (PSM) analysis.

In these reports, the 5‐year overall survival rate of patients with HCC associated with PVTT extending to the first branch or main portal vein was as low as 11% to 21%. Surgical treatment for advanced HCC associated with tumor thrombus extending to the first branch (Vp3) or the main or the contralateral first branch (Vp4) of the portal vein has been reported from Asia, and the 5‐year overall survival rate after liver resection, including removal of tumor thrombus, was reported to be over 20%, suggesting that surgical resection is effective even for cases of highly advanced HCC associated with PVTT.^55^ Although there are limited numbers of reports regarding HCC with hepatic vein tumor thrombus (HVTT), Kokudo et al^56^ carried out a national survey in Japan of the clinical outcome of 1021 such HCC cases. In a PSM analysis, the authors reported that the overall survival rate was significantly better in patients who underwent surgical resection than in those who underwent non‐surgical treatments. HCC associated with bile duct tumor thrombus (BDTT) is uncommon, with an incidence of 2% to 5%, and is characterized by large tumor size, high incidence of PVTT, poor differentiation, and higher TNM stage.^57^ A stage‐matched analysis showed the overall survival of patients with BDTT‐HCC was equivalent to that of those with non‐BDTT‐HCC, suggesting that surgical resection could be appropriate for BDTT‐HCT.^57^, ^58^

#### 3.2.2 Neoadjvant therapy for HCC at an advanced stage

Neoadjuvant therapies for HCC at an advanced stage, including chemotherapy and TACE, have been reported to be effective in reducing tumor size but not in improving prognosis.^59^, ^60^ Many of the published reports are relatively old and the evidence levels of these studies are rather low; many of them are negative regarding neoadjuvant chemotherapy for HCC.

It has been shown that preoperative radiotherapy for HCC associated with PVTT reduced the size of PVTT and showed better progression‐free and survival rates than surgery alone,^61^ suggesting efficacy of preoperative radiotherapy for PVTT.

Although many reports are negative regarding neoadjuvant therapy for HCC, neoadjuvant therapy before liver transplantation showed promising results. TACE^62^ and RFA^63^ have been carried out as downstaging therapies to reduce tumor size to meet the criteria for liver transplantation, and as bridging therapies to maintain criteria until transplantation, showing almost equivalent outcomes to the non‐neoadjuvant therapy group.

#### 3.2.3 Adjuvant therapy for HCC at an advanced stage

After curative resection of HCC, both recurrence from intrahepatic metastasis and de novo HCC can develop. Antiviral therapy has been shown to prevent HCC carcinogenesis in individuals with hepatitis B or C virus infection.^64^, ^65^, ^66^ Even after resection of HBV‐related HCC, antiviral therapy has been reported to be effective in preventing HCC development and improving the survival rate.^67^ Direct‐acting antivirals have been developed against HCV and eradication of HCV is now possible; therefore, the efficacy of adjuvant antiviral therapy for HCV is expected to be reported in the future.

The STORM trial, an RCT investigating the efficacy of sorafenib as an adjuvant therapy for HCC following radical resection or ablation, failed to show the superiority of sorafenib to placebo in recurrence‐free survival (primary endpoint) and in overall survival (secondary endpoint).^68^

Several RCT have been conducted to investigate the efficacy of cytotoxic agents in adjuvant therapy, but none showed promising results. Adjuvant therapy with capecitabine has been reported to significantly prolong the median time to recurrence compared to placebo; however, this therapy has not become standard because the sample size was too small and no significant difference in the survival rate was observed.^69^

## 4 CURRENT TOPICS IN LIVER SURGERY

### 4.1 Laparoscopic liver resection

There is an increasing number of reports regarding the safety and efficacy of operative procedures of laparoscopic liver resection (LLR).^70^, ^71^ During the Second International Consensus Conference for Laparoscopic Liver Resection held in Morioka in 2014, the stages of development of LLR procedures were evaluated in accordance with the Stage of Development According to the Balliol. Minor LLR was at IDEAL stage 3 (“Assessment” phase) whereas major LLR was at IDEAL stage 2b (“Exploration” stage).^72^ Minor LLR has become standard practice and the risks associated with novelty are low. However, continuous assessment of outcomes is encouraged, especially if high‐level studies are lacking. Major LLR is considered to be safe and feasible, but it was judged that there remain risks associated with the newness of the procedure and the lack of experience of surgeons carrying out major LLR.^73^ Recommendation of the consensus meeting was that surgeons undertaking these procedures should be experienced both in liver surgery and advanced laparoscopy and that outcomes should be evaluated in registries and by RCT, where appropriate.^72^ In the International Survey on Technical Aspects of Laparoscopic Liver resection (INSTALL) study, 86.5% of medical institutions responded that the number of LLR cases had increased in the previous 5 years (2009‐2013).^74^

From an oncological point of view, laparoscopic liver resection has been shown to be non‐inferior to laparotomy in many retrospective studies, including those which used PSM to minimize differences in the backgrounds of patients. As shown in Table 3, according to previous meta‐analysis or PSM‐based studies comparing LLR and OLR, LLR is associated with smaller amounts of blood loss, almost similar operative time (although the operative time was reported to be longer in LLR than in OLR in some reports), fewer complications, and shorter hospital stay. Regarding the long‐term treatment outcome for HCC, there were no significant differences in overall survival and DFS between LLR and OLR.^75^, ^76^, ^77^, ^78^, ^79^, ^80^, ^81^ At present, there is no RCT comparing LLR and OLR, but there seems to be no difference in the long‐term outcome between LLR and OLR for HCC. In addition, it is pointed out that LLR may be superior to OLR in patients with impaired liver function.^82^, ^83^ Most patients with HCC have underlying chronic liver disease, and their liver function is often impaired. Therefore, indication for surgery should be considered regarding both the oncological therapeutic effects and the risk of postoperative complications, especially the development of liver failure. Survival outcomes were comparable between LLR and OLR in patients with early‐stage HCC; however, the laparoscopic approach provides a better disease‐free survival rate in patients with advanced stage HCC.^84^ The differences are considered to be caused by low surgical stress, including less blood loss and less tissue manipulation, and so on. The feature of laparoscopic approach will lead to expanding the surgical indications for HCC with a background of chronic liver disease.

**Table 3:** Recent studies regarding outcomes of laparoscopic liver resection vs open liver resection

Author	Year	Type	Blood loss	Operative time	Hospital stay	Morbidity	Surgical margin	Survival	RFS
Zhou et al^75^	2011	Meta‐analysis	LLR < OLR	NSD	LLR < OLR	LLR < OLR	NSD	NSD	NSD
Li et al^76^	2012	Meta‐analysis	LLR < OLR	NSD	LLR < OLR	LLR < OLR	NSD	NSD	NSD
Kim et al^77^	2014	PSM LLR (n = 29) OLR (n = 29)	NSD	NSD	LLR < OLR	NSD	NSD	NSD LLR 5 y 92.2% OLR 5 y 87.7%	NSD LLR 5 y 54.0% OLR 5 y 40.1%
Chen et al^78^	2015	Meta‐analysis	NSD	NSD	LLR < OLR	LLR < OLR	NSD	NSD	NSD
Takahara et al^79^	2015	PSM LLR (n = 387) OLR (n = 387)	LLR < OLR	LLR > OLR	LLR < OLR	LLR < OLR	NSD	NSD LLR 5 y 76.8% OLR 5 y 70.9%	NSD LLR 5 y 40.7% OLR 5 y 39.3%
Sposito et al^80^	2016	PSM LLR (n = 43) OLR (n = 43)	NSD	NSD	LLR < OLR	LLR < OLR	NSD	NSD LLR 5 y 38% OLR 5y 46%	NSD LLR 5 y 25% OLR 5 y 11%
Landi et al^81^	2017	PSM LLR (n = 208) OLR (n = 216)	LLR < OLR	NSD	LLR < OLR	NSD	N/A	N/A	N/A

LLR, laparoscopic liver resection; N/A, not available; NSD, no significant difference; OLR, open liver resection; PSM, propensity‐score matched; RFS, recurrence free survival.

Safety is the primary concern regarding the introduction of laparoscopic liver resection; therefore, guidelines are needed for that purpose. In addition to conventional classification (minor and major liver resection), a scoring system of surgical difficulty on the basis of liver functional reserve and tumor factors, including tumor location and relationship to major vessels, has been proposed in an attempt to serve as a guideline for training,^85^, ^86^, ^87^ and has been validated to correlate with surgical outcome in the clinical setting.^88^, ^89^, ^90^ A step‐by‐step training system appropriate for the difficulty score and individual surgical skill can lead to safe expansion of the indication of LLR.

## 5 CONCLUSIONS

We have reviewed the current topics of HCC treatment, focusing on surgical therapy. Introduction of a new modality for ablation and a new technique for surgery has increased the variety of treatment options customizable for individual patients. However, the increasing complexity of treatment options makes establishment of evidence and interpretation of results from RCT comparing standardized treatments more difficult. The current problem in surgical therapy for HCC is a lack of studies with a high level of evidence quality. Treatment strategy is influenced largely by social background in each area because of the different guidelines used. Because there are differences in the backgrounds of each area, high‐quality studies are expected to be conducted to provide robust evidence, which will be reflected in the guidelines in each area.

## DISCLOSURE

Funding: This work was supported in part by JSPS KAKENHI Grant Number 15H04926 from the Ministry of Health, Labor, and Welfare of Japan.

Conflicts of Interest: Authors declare no conflicts of interest for this article.
